# The importance of allergic disease in public health: an iCAALL statement

**DOI:** 10.1186/s40413-018-0187-2

**Published:** 2018-04-27

**Authors:** Mario Sánchez-Borges, Bryan L. Martin, Antonella M. Muraro, Robert A. Wood, Ioana O. Agache, Ignacio J. Ansotegui, Thomas B. Casale, Thomas A. Fleisher, Peter W. Hellings, Nikolaos G. Papadopoulos, David B. Peden, James L. Sublett, Stephen A. Tilles, Lanny Rosenwasser

**Affiliations:** 1 Allergy and Clinical Immunology Department, Centro Medico Docente La Trinidad, Allergy Service, Clinica El Avila, 6a. Transversal Urb. Altamira, Piso 8, Consultorio 803, Caracas, 1060 Venezuela; 2 Ohio State University, Powell, OH USA; 3 Padua University Hospital, Padua, Italy; 4 Johns Hopkins University, Baltimore, MD USA; 5 Spitalul Judetena Brasov, Brasov, Romania; 6 Hospital Quirónsalud Bizkaia, Bilbao, Spain; 7 University of South Florida Morsani College of Medicine, Tampa, FL USA; 8 National Institutes of Health, Bethesda, MD USA; 9 KU Leuven, Leuven, Belgium; 10 University of Athens, Athens, Greece; 11 University of North Carolina, Chapel Hill, NC USA; 12 Family Allergy and Asthma, Louisville, KY USA; 13 Northwest Asthma and Allergy Center, Redmond, WA USA; 14 University of Missouri School of Medicine, Kansas City, MO USA

## Introduction

Allergic diseases constitute a significant cause of morbidity worldwide and a considerable burden on the health and medical systems of both developed and emerging economies. Allergies and related diseases including asthma, rhinosinusitis, atopic dermatitis and life threatening food, drug, and stinging insect allergies affect at least 30% of the population and nearly 80% of families. According to recent studies, their prevalence is increasing globally [1–4].

Medical services providing expert allergy care are lacking in many countries; therefore, the major organizations devoted to the field of allergy (American Academy of Allergy, Asthma and Immunology, AAAAI; American College of Allergy, Asthma, and Immunology, ACAAI; European Academy of Allergy, Asthma and Clinical Immunology, EAACI; and the World Allergy Organization, WAO), strongly feel that education of health professionals and the public on the importance and impact of allergic diseases as a public health concern should be encouraged.

The International Collaboration in Allergy, Asthma, and Immunology (iCAALL), a partnership constituted by AAAAI, ACAAI, EAACI, and WAO, have recommended publishing an advocacy statement with the purpose of calling to the attention of the medical community, health authorities and the public in general, the major impact and relevance of the allergy specialists as key groups of professionals specifically trained for the adequate diagnosis, treatment, and prevention of allergic diseases.

### Allergic diseases are often underdiagnosed and undertreated

Allergy is a rather “new” medical specialty, having only emerged with increased awareness of immunologic responses and the increasing importance of non-communicable diseases. However, in spite of being a major global public health issue, the public and the health establishment have generally not recognized the importance of allergic diseases. Since the prevalence of allergic diseases has been steadily increasing, it is time to place the field of allergy in a more prominent place within global medical organizations.

This absence of proper recognition frequently results in a lack of, or incorrect, diagnosis resulting in sub-optimal disease management, negative effects on quality of life, increased morbidity and mortality, and considerable additional direct and indirect costs. Moreover, the complexity and involvement of multiple organs and systems of allergic diseases confounds management in fragmented care based on our current health care delivery systems dependent on traditional organ-based specialists.

### Unmet needs in allergic care

In most populations around the world there is a lack of adequate education on the definition, etiology, pathogenesis, proper therapies, and prevention of allergic diseases. Awareness of the morbidity and potential mortality associated with allergic diseases, the chronic nature of those conditions, and the importance of consulting a physician knowledgeable in allergic diseases, asthma and clinical immunology are often lacking. This translates into patients with allergic diseases not being managed by physicians with the necessary training and skills in the appropriate use of efficacious medications required for optimal management.

There is an increasing need to expand the number of allergy/clinical immunology specialists as well as local and regional diagnostic and treatment centers in order to facilitate timely referrals for patients with complex allergic diseases. A goal should be established to guarantee the universal accessibility to affordable and cost-effective therapies as well as novel medications used in the management of allergic diseases.

Presently, there are millions of people worldwide who do not have access to care by specialists in allergy [1]. Moreover, epinephrine auto-injectors, some drugs for severe asthma, allergen-specific immunotherapy, and some drugs for adverse reactions to biologicals and chemotherapeutic agents in desensitization centers are not available in many parts of the world.

Public health officers should provide for optimal allergy/clinical immunology services, including access to specialists and diagnostic and treatment centers. Allergists should be able to prescribe the most cost-effective medications to manage the specific clinical findings of each allergic patient.

Consultations with allergists to assure correct diagnosis and treatment are indispensable to improve long-term patient outcomes and their quality of life and reduce the unnecessary additional direct and indirect costs passed on to the patient, payer and society.

In the last decade there have been important advances in the field of allergy, especially in the understanding of the mechanisms leading to disease, improved diagnostic methods based in molecular allergy, and novel medications for immune modulation and immunotherapy based in more effective and safe vaccines and biologicals [5–9].

### What can be done to improve the current situation?

Advocacy from these professional organizations on the role of allergy in public health has been identified as the most important objective of iCAALL. Increasing awareness on the relevance of allergic diseases as a major public health problem could lead to a better recognition by governments and health authorities.

Programs to increase awareness of allergic diseases should focus on the causes, prevention, control, and economic impact. The main goal would be better care of allergic diseases around the world with the aim to effectively engage regional, country, and local authorities.

An important step forward supported by the major allergy organizations resides in current efforts to obtain World Health Organization (WHO) recognition of allergy through the new nomenclature of allergic diseases included in the ICD-11 classification of diseases [10, 11].

Because allergic diseases are systemic multi-organ diseases, allergists are in the best position to diagnose and manage the allergic patient, in contrast to the classic organ-based approach of most other medical specialties. Allergic disease management by allergists based on the new technological advancements could be more efficacious and cost-effective when compared to the care provided by generalists or other specialists.

In addition, allergists have significant experience in the implementation of preventive measures which have been shown to diminish or eliminate allergic symptoms, including environmental control measures and allergen-specific immunotherapy, which could reduce the costs of disease management.

### Focus on the patient

Educational efforts focused on allergic diseases should be specifically directed to patients and their families as the final targets of these awareness programs. In order to obtain better results regarding disease prevention and control, it will be strictly necessary to reach the community at various levels, including regional, local, and state agencies as well as schools and patient organizations with straight forward and understandable messaging.

At the same time, approaching governments, politicians and public health officers should be part of the strategy in order to promote the allocation of sufficient resources for the diagnosis, control, treatment and prevention of allergic diseases.

#### Notes

This is a Joint Statement by the organizations of the International Collaboration on Allergy Asthma and Immunology (iCAALL): American Academy of Allergy Asthma and Immunology (AAAAI), represented by Robert Wood, Thomas Casale, Thomas Fleisher, David Peden); American College of Allergy Asthma and Immunology (ACAAI), represented by Bryan Martin, James L Sublett, Stephen A Tilles; European Academy of Allergy Asthma and Clinical Immunology (EAACI), represented by Antonella Muraro, Ioana Agache, Peter Hellings, Nikos Papadopolous; World Allergy Organization (WAO), represented by Mario Sánchez-Borges, Ignacio J Ansotegui, Lanny Rosenwasser. Published on behalf of AAAAI, ACAAI, EAACI, and WAO.

## Abbreviations

iCAALL: International Collaboration in Allergy Asthma and Immunology
